# Long-term antiplatelet monotherapy after PCI: searching for the smart choice

**DOI:** 10.1093/ehjcvp/pvaf032

**Published:** 2025-04-26

**Authors:** Felice Gragnano, Vincenzo De Sio, Mattia Galli, Paolo Calabrò

**Affiliations:** Department of Translational Medical Sciences, University of Campania ‘Luigi Vanvitelli’, Viale Abramo Lincoln 5, Caserta IT-81100, Italy; Division of Clinical Cardiology, A.O.R.N. ‘Sant’Anna e San Sebastiano’, Caserta, Italy; Department of Translational Medical Sciences, University of Campania ‘Luigi Vanvitelli’, Viale Abramo Lincoln 5, Caserta IT-81100, Italy; Division of Clinical Cardiology, A.O.R.N. ‘Sant’Anna e San Sebastiano’, Caserta, Italy; GVM Care & Research, Maria Cecilia Hospital, Cotignola, Italy; Department of Medical-Surgical Sciences and Biotechnologies, Sapienza University of Rome, Latina, Italy; Department of Translational Medical Sciences, University of Campania ‘Luigi Vanvitelli’, Viale Abramo Lincoln 5, Caserta IT-81100, Italy; Division of Clinical Cardiology, A.O.R.N. ‘Sant’Anna e San Sebastiano’, Caserta, Italy

Determining the optimal long-term antiplatelet regimen for secondary prevention after percutaneous coronary intervention (PCI) remains a matter of debate, especially in patients at high risk of adverse events.^1-3^ Historically, aspirin monotherapy after a defined course of dual antiplatelet therapy (DAPT) has been the standard of care; however, its primacy has been challenged. In a meta-analysis, P2Y_12_ inhibitor monotherapy was superior to aspirin for anti-ischaemic protection, with patients from Asian cohorts appearing to derive greater benefit than those from Europe and North America, particularly for stroke prevention.^4^

SMART-CHOICE 3, a rigorously conducted multicenter, randomized, open-label trial involving 5542 patients from South Korea, provides new evidence in the clinical arena.^5^ Targeting a high-risk post-PCI population of patients with prior myocardial infarction, medication-treated diabetes, or complex coronary lesions, who had already completed a standard period of DAPT (≥12 months for myocardial infarction, ≥6 months for other indications), the study compared clopidogrel and aspirin monotherapy for chronic treatment. Over a median follow-up of 2.3 years, clopidogrel monotherapy significantly reduced the incidence of the composite primary endpoint (all-cause death, myocardial infarction, or stroke) compared with aspirin [4.4% vs. 6.6%, hazard ratio (HR) 0.71, 95% confidence interval (CI) 0.54–0.93; *P* = 0.013]. This benefit was primarily driven by a reduction in myocardial infarction (1.0% vs. 2.2%, HR 0.54, 95% CI 0.33–0.90). No difference was observed in bleeding outcomes. These findings extend the evidence from the HOST-EXAM trial, focusing on a more enriched population for ischaemic risk and on hard clinical endpoints.^4^ However, the open-label design and exclusive enrolment of East Asian patients may limit the generalizability of the results.^5^ Of note, up to 30% of individuals treated with clopidogrel are less responders to this agent, a phenomenon associated with increased thrombotic risk.^6^

In conclusion, in the SMART-CHOICE 3 trial, clopidogrel monotherapy was more effective as a long-term strategy than aspirin in PCI patients at high ischaemic risk who have completed their course of DAPT. Further trials involving non-Asian populations and incorporating assessments of clopidogrel responsiveness are warranted to better define the implications of these findings in broader clinical settings.

## Data Availability

The data underlying this article were taken from the quoted references.
